# Emergency care in the pandemic

**DOI:** 10.2471/BLT.20.021020

**Published:** 2020-10-01

## Abstract

Optimizing emergency care during what may be a protracted pandemic will require more than supplies, infrastructure, training and support for health workers. Lynn Eaton reports.

When the first novel coronavirus disease (COVID-19) cases started to show up in Sudan in March, Dr Yasein Omer was concerned about how the country’s health workforce would respond.

A final year registrar of emergency medicine affiliated with the Sudan Medical Specialisation Board, Omer rotates among the state-run hospitals in the capital, Khartoum.

“Most of the health workers were scared of COVID-19,” he recalls, “but the doctors and nurses in the emergency departments knew what they had to do, and they really took the weight on their shoulders.”

Over the past six months or so, emergency department staff have been doing the same thing all over the world: caring for people with COVID-19, often without adequate resources including personal protective equipment.

And their efforts have been applauded – literally in some countries, with people banging pots and pans at agreed times in recognition of their service.

“COVID-19 has really put the people who deliver emergency care in the spotlight,” says Dr Teri Reynolds, an expert in emergency, trauma and acute care at the World Health Organization (WHO), “but the pandemic has also highlighted the long-standing absence of capacity and organization in this key area.”

Despite a World Health Assembly resolution in 2019, calling for Member States to ensure timely care for the acutely ill and injured as a vital component of universal health coverage, emergency care systems continue to be under resourced and are inadequate in most countries.

“Perhaps one of the greatest collective hallucinations is that all hospitals have emergency departments,” Reynolds says.

COVID-19 may be dispelling that assumption, as health authorities confront their systems’ limits, especially their inability to cope with sudden surges in demand.

In response, many governments have ploughed resources into emergency care, setting up field hospitals to increase the number of available beds, and scrambling for medical equipment on international markets.

“One of the greatest collective hallucinations is that all hospitals have emergency departments.”

Teri Reynolds

International organizations and donors have committed resources to supporting lower income countries in their efforts to ramp up capacity. For example, in April the World Bank Group committed to deploying up to US$ 160 billion over 15 months to help countries respond to immediate health consequences of the pandemic and bolster economic recovery.

Ethiopia is one country to have received some of these resources – US$ 82 million to help address critical needs for COVID-19 preparedness and response, including the provision of medical equipment, health system capacity building, and support to establish treatment centres.

Dr Alegnta Gebreysesus, Ethiopia’s director for emergency and critical care services, welcomes the injection of funds as well as more attention to emergency care advocates like herself. “COVID-19 has given us a unique opportunity to point out to all stakeholders what emergency care is and why it is so important,” she says.

While Reynolds and her colleagues at WHO also welcome the increased focus on emergency care, they are concerned that capacity-building efforts related to COVID-19 may be too narrowly targeted.

“There has been a lot of focus on the care required to deal with the most critical cases of respiratory distress,” says Dr Pryanka Relan, a Technical Officer working on WHO’s COVID-19 Clinical Management Team. “Much of that focus has been on ventilators,” she adds, referring to the devices used to deliver oxygen and support breathing.

Ventilators have become something of a policy emblem in recent months, with politicians citing the number of ventilators they have been able to acquire as a measure of the strength of their COVID-19 response.

“While it’s understandable that people focus on the gap in basic critical care resources, it’s important to realize that ventilation is only required in approximately 5% of hospitalized cases. Much can and should be done for patients before mechanical ventilation with intubation becomes necessary,” Relan says.

Reynolds concurs, arguing that optimizing emergency care delivery depends largely on actions taken before critical care becomes necessary. “It’s a question of catching cases as early as possible and then making sure patients are properly triaged and referred,” she says.

Catching people early makes it possible to isolate people early and intervene early, for example by providing oxygen supplementation through nasal cannulae. “By intervening early you can actually prevent people from ending up on ventilators,” says Relan.

Early reports from Lombardy in northern Italy, where Europe’s COVID-19 outbreak first took hold, suggest that about 50% of patients given oxygen with non-invasive continuous positive airway pressure avoided the need for invasive ventilation.

WHO guidance and COVID-19 training on screening, isolation, triage and referral recommends that the process begin at the first point of contact, which can be with community health workers, at community screening sites, clinics, health posts, pharmacies or on the phone.

“Basically, the earlier you identify sick people, the better,” says Reynolds, “and then you need effective triage because that’s the only way to cope with surges, and direct medical resources to support the critically ill and protect the safety of health-care workers.”

The importance of screening and triage is well understood by clinicians on the front line.

“You cannot begin to look at emergency care in a hospital if the whole system is not set up appropriately, and that includes effective screening and triage,” argues Dr Ken Diango, a doctor from the Democratic Republic of the Congo, who is currently pursuing PhD studies in Emergency Medicine at the University of Cape Town, South Africa.

Diango hopes that COVID-19 will spur efforts to introduce health-system-wide reform in his country including expanding coverage of emergency care. “Covid-19 has laid bare the gaps in the emergency-care system in DRC and without proper emergency care, the goal of universal health coverage will not be possible,” he says.

Dr Hendry Sawe, President of the Emergency Medicine Association for the United Republic of Tanzania, is similarly convinced of the importance of effective triage.

“Without proper emergency care, the goal of universal health coverage will not be possible.”

Ken Diango

“In the past in Tanzania, people would just turn up at the hospital and care would be based on first come, first served, the same as if you were going to the bank,” he says. “We now offer training programmes for emergency medicine doctors and nurses, based on WHO guidance, which includes guidance on effective triage.”

While he is proud of what has been achieved in the United Republic of Tanzania over the past ten years, Sawe, like Diango, has no illusions about the challenges faced in his country, saying that for the 70% of the population living in rural areas, an emergency department in Dar es Salaam has limited impact.

For Reynolds, optimal allocation of resources through effective screening and triage will only become more important as the pandemic progresses.

“There have been several statements, regarding the likelihood of this being a protracted pandemic, and if there is a significant increase in cases later in the year, and it proves difficult to impose the kinds of lockdowns we saw back in March, health systems are going to struggle unless they get better organized,” she says.

Nowhere is this truer than in resource-poor countries. “The less you have, the more important it is to use resources wisely and that includes getting the right people to the right resources in a timely fashion,” Reynolds says.

WHO has been working with national governments through the WHO Global Emergency and Trauma Care Initiative. The Sudanese government asked for guidance on setting up a COVID-19 response and has been training staff on the use of WHO emergency care tools including the *Interagency Integrated Triage Tool* and *Basic Emergency Care.*

In June, the European Union committed € 10 million (US$ 11.5 million) to a project designed to support that response and improve the country’s overall health system. Implemented by WHO in support of Sudan’s Federal Ministry of Health, the two-year project will cover health preparedness needs across the country, including coordinating the emergency response, ramping up surveillance and testing, and isolating and managing COVID-19 patients. Ten isolation centres with ICU beds, oxygen, ventilators and other equipment will be established, and basic water services will also be set up in 12 hospitals.

“We are hoping that the lessons we are learning now, and the investments that are being made in our health system as a whole, will result in a more resilient health system,” Omer says.

It is a hope Reynolds shares. “If there is a silver lining in the pandemic it is perhaps the fact that emergency health is starting to get the resources it deserves. Because emergencies happen all the time and to respond to them, we need strong, resilient emergency care systems.”

**Figure:**
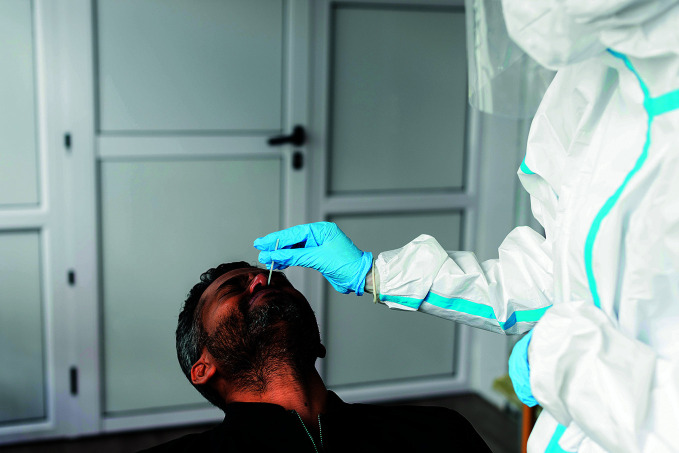
Testing for COVID-19 at the Jawaharlal Nehru Hospital in Rose Belle, Mauritius.

WHO/Blink Media/Gilliane Soupe

**Figure:**
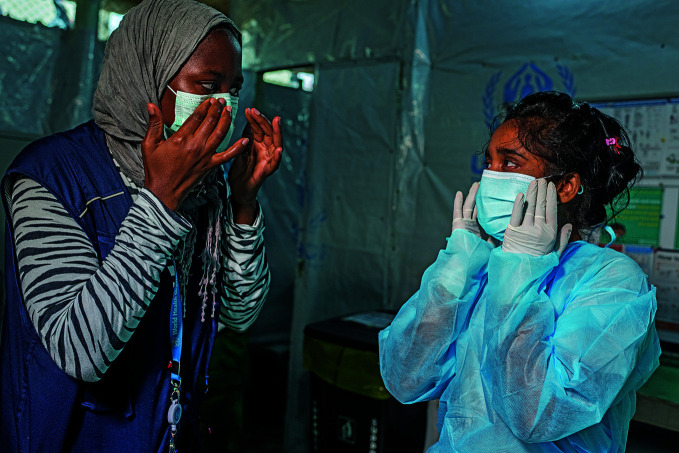
A WHO Infection Prevention and Control Specialist helps a nurse put on personal protective equipment at a clinic in a Rohingya camp in Cox’s Bazar, Bangladesh.

WHO/Blink Media Fabeha Monir

